# Arrangements with Herbalists

**Published:** 1913-05-10

**Authors:** Gordon P. C. Ward

**Affiliations:** National Medical Guild. 34 Villiers Street, Strand, W.C.


### Arrangements with Herbalists.

*To the Editor of* The Hospital.

Sir,—You kindly published recently a letter from the National Medical Guild in which attention was called to the fact that treatment by quacks, herbalists, and others was being permitted under the " own arrangements " clause of the National Insurance Act.

May we be further permitted to state that the London Insurance Committee is prepared (on the evidence of its Chairman, given in the House of Commons recently) to allow arrangements to be made with " homœopaths and herbalists," and this in spite of the fact that Form 43 I.C., on which application to make " own arrangements " must be made, contains the statement that " the Committee will also require to be satisfied that the treatment obtained will be of a nature, quality, and extent not inferior to that obtained under the panel system ? "

The Parliamentary representative of the Insurance Commissioners—namely, Mr. Masterman—has made it clear that the matter is one for the discretion of the Committees, and one which he did not feel disposed to take up.

Seeing that this has been a bone of contention in Germany and other Continental countries, it is well that the profession in England should take the matter in hand as early as possible, and the first step to this is to understand it fully.

We would, therefore, ask you to be so good as to give publicity to this letter.—We are, Sir, yours faithfully,

Gordon P. C. Ward,
National Medical Guild.

34 Villiers Street, Strand, W.C.,
May 6, 1913.

